# Enhanced Electrochemical performance at high temperature of Cobalt Oxide/Reduced Graphene Oxide Nanocomposites and its application in lithium-ion batteries

**DOI:** 10.1038/s41598-018-37032-5

**Published:** 2019-01-10

**Authors:** Yasmin Mussa, Faheem Ahmed, Hatem Abuhimd, Muhammad Arsalan, Edreese Alsharaeh

**Affiliations:** 1 College of Science and General Studies, Alfaisal University, P.O. Box 50927, Riyadh, 11533 Saudi Arabia; 2 National Nanotechnology Center, King Abdulaziz City for Science and Technology, P.O. Box 6086, Riyadh, 11442 Saudi Arabia; 3 EXPEC Advanced Research Center, Saudi Aramco, P.O. Box 5000, Dhahran, 31311 Saudi Arabia

## Abstract

We report a microwave irradiation method for the preparation of reduced graphene oxide (RGO) based Co_3_O_4_ nanocomposites as anodes for lithium-ion (li-ion) batteries. The Co_3_O_4_/RGO nanocomposites displayed good electrochemical behavior as anodic materials for li-ion batteries when compared to pure Co_3_O_4_. The Co_3_O_4_/RGO nanocomposites with low RGO content resulted in stable electrochemical performance with 100% coulombic efficiency at a high current density of 500 mA/g for 50 cycles. The enhanced capacity of the Co_3_O_4_/RGO nanocomposites is due to the incorporation of RGO, which resulted in a four times larger surface area than that of Co_3_O_4_. This increased surface area could facilitate the absorption of more lithium ions, resulting in excellent electrochemical performance. Interestingly, the novelty of this work is that the designed li-ion batteries showed stable electrochemical performance even at a high temperature of 100 °C, which might be useful for rechargeable battery applications in a wide temperature range.

## Introduction

Lithium-ion batteries have a number of applications as energy storage units mainly in electric vehicles and electronic devices owing to their high capacity, long cycling life, and environmental friendliness^1^. There is an urgent need to improve these batteries to meet energy demand requirements^2^. One method to enhance li-ion batteries perfomance is to design alternative negative or anode materials, including transition metal oxides, and replace the conventional anode material, graphite, which has a theoretical capacity of only 372 mAh/g^3^.

Currently, transition metal oxides have received considerable attention as anodic materials for use in li-ion batteries^4^ because of their superior theoretical capacity, with approximately three times the capacity of the commercial graphite that have a capacity of 372 mAh/g, making these materials good candidates for energy storage systems. Among the transition metal oxides, Co_3_O_4_ is the most frequently used as anode materials because of its high theoretical capacity (890 mAh/g)^5^.

Generally, cobalt oxides, that include binary oxides such as Cobalt (II) oxide (CoO), Cobalt (III) oxide (Co_2_O_3_) and Cobalt (II, III) oxide (Co_3_O_4_), have been widely explored for applications in li-ion batteries. However, Co_3_O_4_ is synthesized more easily than the other two, as it can be prepared from different cobalt salts by heating in air at 300 to 400 °C^6^. However, one limitation of Co_3_O_4_ nanoparticles as anodic material for li-ion batteries is that they experience poor cycling stability and irreversible capacity loss due to the volume expansion/contraction and agglomeration of the Co_3_O_4_ nanoparticles^7–9^.

One way to solve the above issues is to synthesize Co_3_O_4_ nanoparticles with carbon, such as graphene, which can also improve the conductivity of Co_3_O_4_^10,11^. Graphene has a high surface area, good mechanical properties, and high electrical conductivity, which helps in improving the electrochemical properties of metal oxides^12^.

Many synthetic routes to prepare Co_3_O_4_ nanoparticles have been have been reported, such as co-precipitation, hydrothermal synthesis, thermal decomposition, and reduction^13–15^. Whereas, *in situ* reduction of cobalt salt in the presence of graphene oxide is commonly followed to prepare Co_3_O_4_/RGO nanocomposites^16^. The structural properties of a material, which includes porosity and surface area, strongly affects their performance as electrodes in li-ion batteries making it challenging to design electrode materials.

In contrast to the above mentioned conventional synthesis methods, microwave-assisted techniques of electrode materials for li-ion batteries can provide easy, fast and large-scale synthesis of nanomaterials, in addition to, controllable parameters and energy saving characteristics^17^. In microwave irradiation technique, heating occurs via two mechanisms namely, polarization and conduction. In polarization process, materials are directly heated by radiation, and the radiation or external electric field interacts with the polar molecules that possess a dipole moment and are forced to reorient by rotation which leads to collision and heat generation. However, to generate heat, a substance must possess a dipole moment such as a water molecule this is because external electric fields are sensitive to dipole. In conduction mechanism, heat is generated via the collision of ions in the sample with the neighboring atoms or moleucles^17,18^.

Sun *et al*., conducted a survey on microwave irradiation’s effect and the size and shape of graphene based nanocomposites on their electrochemical performances^17^. Of the different morphologies, graphene based 2D transition metal oxides is a favorable morphology as it assists in facilitating li-ion diffusion and other^17^. Microwave irradiation is favorable for the 2D growth of inorganic nanocrystals, and as reported in our previous study, porous 2D Co_3_O_4_/RGO nanocomposites were obtained via microwave-assisted method making it potential candidate for li-ion batteries^19,20^.

Many research groups have reported the application of graphene-based Co_3_O_4_ nanocomposites as anodic materials in li-ion batteries, and in most of the reported studies, the designed li-ion batteries displayed high specific capacity and stable performance only with high RGO content^21^ and low current densities at room temperature or in a narrow operating temperature range.

In this study, to overcome the issue of the narrow operating temperature range of li-ion batteries, Co_3_O_4_/RGO nanocomposites with a low RGO content were prepared through a microwave irradiation-assisted solution route and used for high-temperature rechargeable batteries with high electrochemical perfomance and good thermal stability.

## Experimental Details

### Preparation of Co_3_O_4_ nanoparticles and Co_3_O_4_/RGO nanocomposites

Co_3_O_4_ nanoparticles and Co_3_O_4_/RGO nanocomposites were synthesized by a microwave irradiation-assisted solution method following the procedures described in our previous work^22^.

### Material characterization

The chemical compositions of the samples were determined using Fourier transform infrared spectroscopy (FTIR, Thermo Scientific Nicolet-iS10) recorded in the range of 4000–400 cm^−1^. A thermogravimetric analyzer (TGA, STA7200) was used to determine the thermal stability of the materials from ambient to 500 °C at a heating rate of 5 °C/min under nitrogen atmosphere. A transmission electron microscopy (TEM, JEOL JEM-2100F) was used to study the morphology of the materials. The Brunauer–Emmett–Teller (BET) was used to obtain the specific surface area through a surface area analyzer (Micromeritics ASAP 2020) by N_2_ adsorption-desorption while the pore size distribution was determined by the Barrett–Joyner–Halenda (BJH) method.

### Electrochemical characterization

To fabricate the working electrode, the active material (80%) was mixed with a conductive agent carbon black (10%) and a binding agent polyvinylidene fluoride (PVDF) (10%) in 50:50 ethanol:dimethylsulfoxide (DMSO) to form a homogenous slurry followed by casting them onto copper substrates and drying at 80 °C under vacuum to remove the solvent. The resulting material was then punched to form disks of ~15 mm with an electrode thickness of 50 µm. The specific capacity and current density were obtained based on the mass of the electrodes which is approximately 1 mg. Polypropylene membrane Celgard 2325 was employed as the separator, 1 M LiPF_6_ was used as the electrolyte and lithium as the counter electrode which were then assembled into CR2032 coin-type cells in an argon-filled glove box. Charge/discharge measurements, electrochemical impedance spectroscopy (EIS) and cyclic voltammetry (CV) were all studied using an electrochemical workstation (Gamry 3000). CV was performed in the voltage window from 0 to 3 V at 50 mV/s scan rate. EIS was performed by using a sine wave of 10 mV in a frequency range of 1 Hz –100 kHz. Galvanostatic charge/discharge tests were evaluated in the voltage window from 0 to 3 V. For high temperature testing; the cell was kept inside a bomb calorimeter vessel by connecting the positive and negative terminal of the coin cell battery to the two electrodes of the vessel. The cell was then left inside the vessel at the specified testing temperature for several hours to reach thermal equilibrium. The measurements were performed at 100 °C and were tested by the Gamry potentiostat/galvanostat connected to the bomb calorimeter vessel. The batteries were cycled in a range of 0 and 3 V at 500 mA/g for 50 cycles. CV was also performed in the potential window from 0 to 3 V and at a scan rate of 50 mV/s. EIS was conducted at a frequency range of 1 Hz – 100 kHz by using a sine wave of 10 mV.

## Results and Discussion

**Figure 1:**
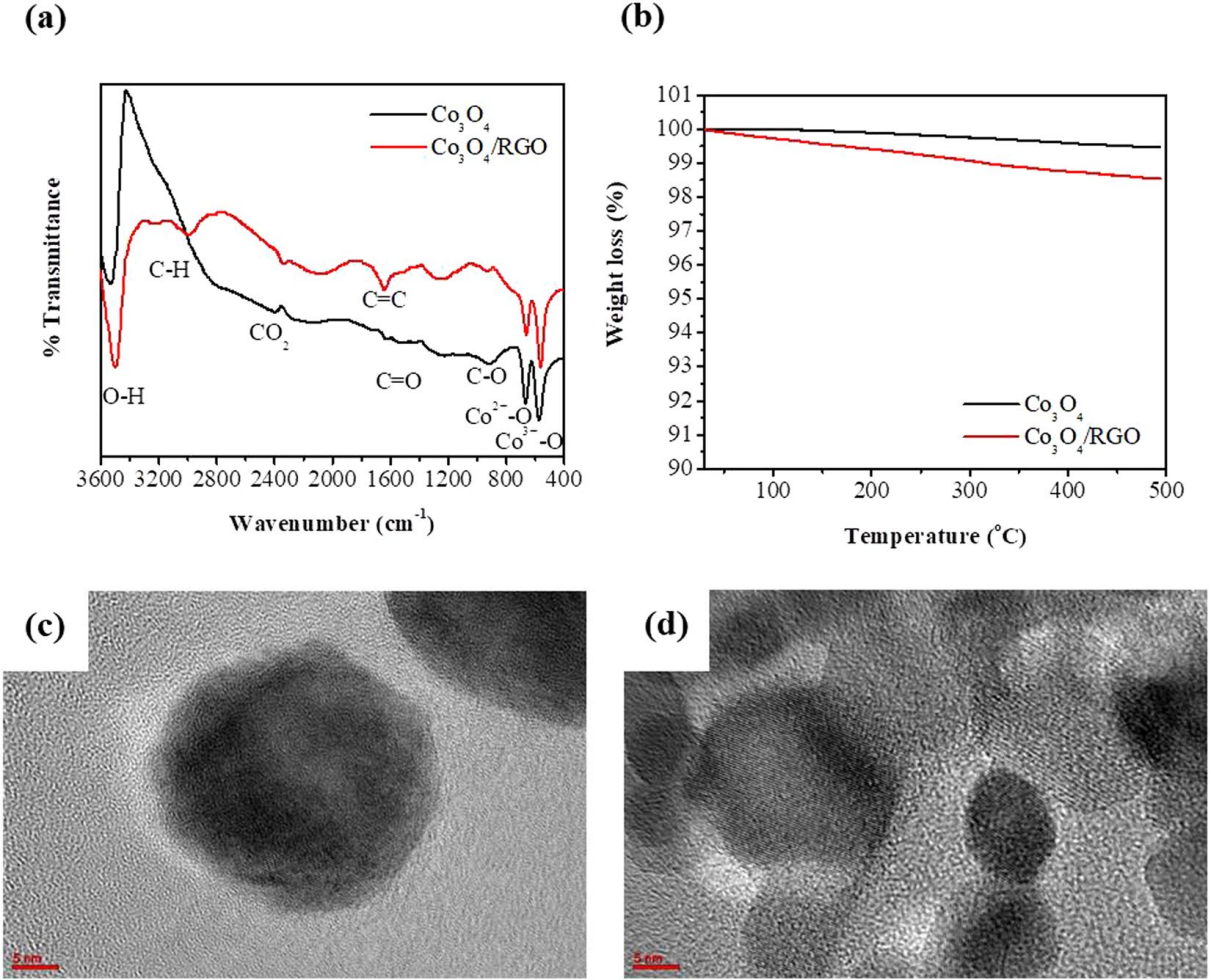
(**a**) FTIR spectra of Co_3_O_4_ and Co_3_O_4_/RGO nanocomposites, (**b**) TGA curves of Co_3_O_4_ and Co_3_O_4_/RGO nanocomposites, (**c**) TEM of Co_3_O_4_ and (**d**) Co_3_O_4_/RGO nanocomposites.

FTIR was used to demonstrate the effect of RGO on the chemical structure of the Co_3_O_4_ nanoparticles. Fig. 1(a) shows the FTIR spectra of Co_3_O_4_ nanoparticles and Co_3_O_4_/RGO nanocomposites. The absorption bands at 586.61 cm^−1^ and 671.05 cm^−1^ are assigned to Co-O stretching vibrations and O-Co-O bridging vibrations, respectively. For the pure Co_3_O_4_ nanoparticles, the weak absorption bands at 1052.41 cm^−1^ and 1251.76 cm^−1^, are assigned to C-O stretching vibrations, the band at 1649.67 cm^−1^ and the C-H bands near 2980.64 cm^−1^ are due to the presence of cobalt acetate (Co(CH_3_COO_2_)) residues. However, the presence of the O-H band at 3526.79 cm^−1^ suggests the possibility of adsorbed water. For the RGO-based nanocomposites, some functional groups were still detected. Although the peaks decreased and almost disappeared after the reduction of GO to RGO due to the deoxygenation process, in this case, many peaks were detected due to the partial reduction of GO. Two peaks at 856.9 cm^−1^ and 1118.05 cm^−1^ are attributed to the alkoxy and epoxy (C-O) groups, respectively, of GO. The peaks at 1636.34 cm^−1^ and approximately 2932.70 cm^−1^ are assigned to the bending of the C=C aromatic rings of RGO and aliphatic C-H groups, respectively. The existence of the C=C peak in the spectra of all the RGO-based samples suggests that the sp^2^ structure of the carbon atoms was retained. The strong C=C peak observed for Co_3_O_4_/RGO indicates a stable graphene structure. Furthermore, the peak centered at approximately 3533.27 cm^−1^ is attributed to the hydroxyl (OH) groups of GO, as GO is considered to be hydrophilic^23–25^.

The weight percentage of RGO in the Co_3_O_4_/RGO nanocomposites and the thermal properties were investigated using TGA. The TGA curves of the Co_3_O_4_ nanoparticles and their RGO nanocomposites from ambient to 550 °C are shown in Fig. 1(b). The TGA plot for the Co_3_O_4_ nanoparticles shows a weight loss of only 0.6% in a single step. This loss might be due to dehydroxylation of the β-Co(OH)_2_ species generated during the synthetic process. This result indicates that the Co_3_O_4_ nanoparticles are thermally stable with no dramatic mass loss. Based on the weight losses, the TGA curve of the RGO based nanocomposites can be divided into three. In the region from ambient to 100 °C, a clear minor loss of 0.3% is observed for Co_3_O_4_/RGO, which is caused by the desorption of physisorbed water. In the second region from 100 to 300 °C, an additional weight loss of 0.7% is observed. The loss in this region results from the decomposition of labile oxygen groups, which includes carboxylate, anhydride, lactone, and epoxy or hydroxyl groups, present in RGO. In the region beyond 300 °C, a weight loss of 0.5% is observed, which results from the decomposition of more stable groups, including carbonyl, phenol, and quinine groups. The total loss of 1.5% shows that all the oxygen-containing groups of GO were converted to RGO during the reduction process. According to the mass remaining after 500 °C, the weight percentage of Co_3_O_4_ in the Co_3_O_4_/RGO nanocomposites was estimated to be 99%^26^.

Morphological studies were performed on both the Co_3_O_4_ nanoparticles and Co_3_O_4_/RGO nanocomposites using TEM, as shown in Fig. 1(c,d), respectively. Previously, we reported that the Co_3_O_4_ nanoparticles exhibited nanoporous structures^27^. Using TEM, detailed structures of the Co_3_O_4_ nanoparticles and Co_3_O_4_/RGO nanocomposites were observed; although pores were not clear, the presence of Co_3_O_4_ incorporated on the RGO sheets was observed. In addition to the morphology, the d-spacing of the nanostructures could also be obtained using TEM. The TEM image of a single pure Co_3_O_4_ nanoparticle reveals that it has a spherical shape, as shown in Fig. 1(c). Atomic-resolution TEM of the Co_3_O_4_/RGO nanocomposites clearly shows the Co_3_O_4_ nanoparticles and RGO sheets, as shown in Fig. 1(d). The TEM shows a clear interlayer distance of 0.24 nm, that matches to the (311) plane of fcc Co_3_O_4_ crystals. Also, the interlayer spacing of 0.35 nm, indexed to the (002) plane of RGO, is observed in the TEM image of Co_3_O_4_/RGO, which matches the results obtained from X-ray diffraction (XRD)^28^.

**Figure 2:**
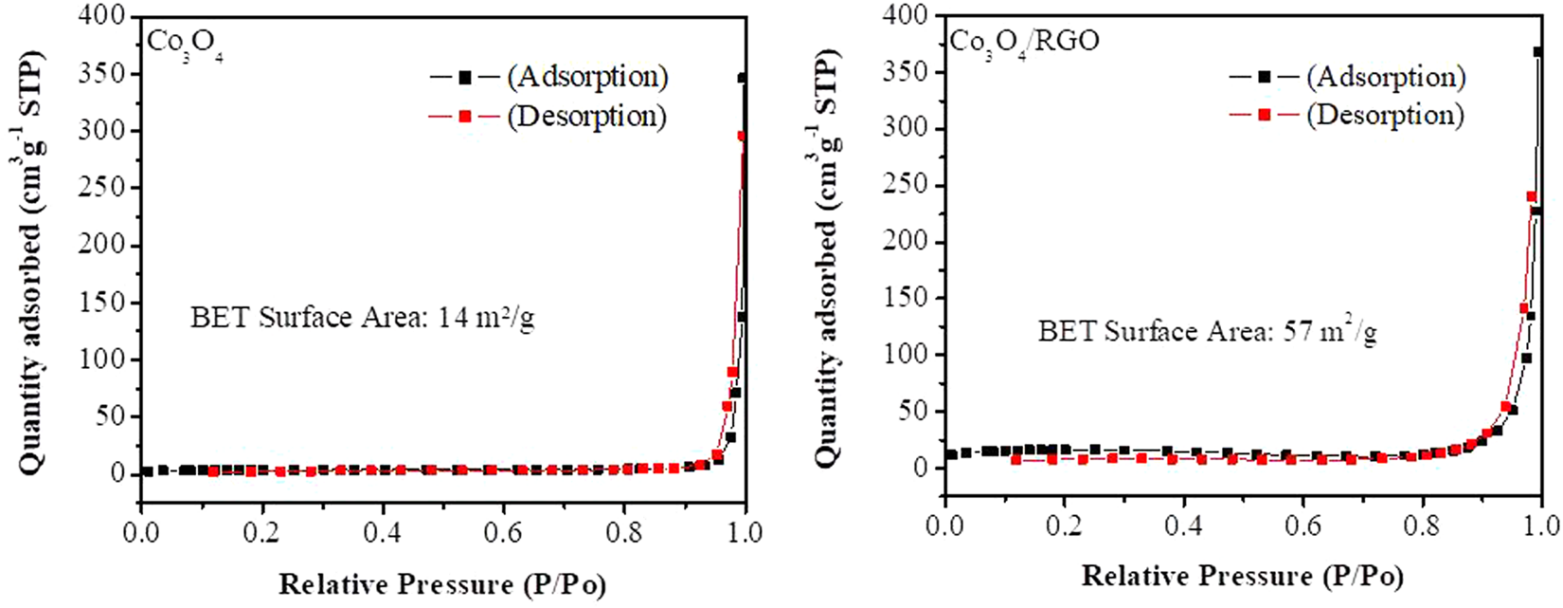
Nitrogen adsorption-desorption isotherms of Co_3_O_4_ and Co_3_O_4_/RGO nanocomposites.

The specific surface area was obtained from the BET isotherms that is a plot of the amount of gas adsorbed as a function of the relative pressure. On the other hand, a plot of pore volume versus pore size gives the pore size distribution and can be calculated using the BJH method. Both the Co_3_O_4_ nanoparticles and Co_3_O_4_/RGO nanocomposites exhibited adsorption–desorption isotherms with typical type IV hysteresis loops, a characteristic of mesoporous materials that have different pore sizes as shown in Fig. 2. Co_3_O_4_/RGO nanocomposites displayed a high BET specific surface area of 57 m^2^/g, which is four times higher than Co_3_O_4_ nanoparticles with a BET specific surface area of only 14 m^2^/g; this increase in the specific surface area is attributed to the addition of RGO to the Co_3_O_4_ nanoparticles. Furthermore, BJH calculations showed that the pore size distributions of the Co_3_O_4_ nanoparticles and Co_3_O_4_/RGO nanocomposites were found to be in in the range of 80–90 nm.

**Figure 3:**
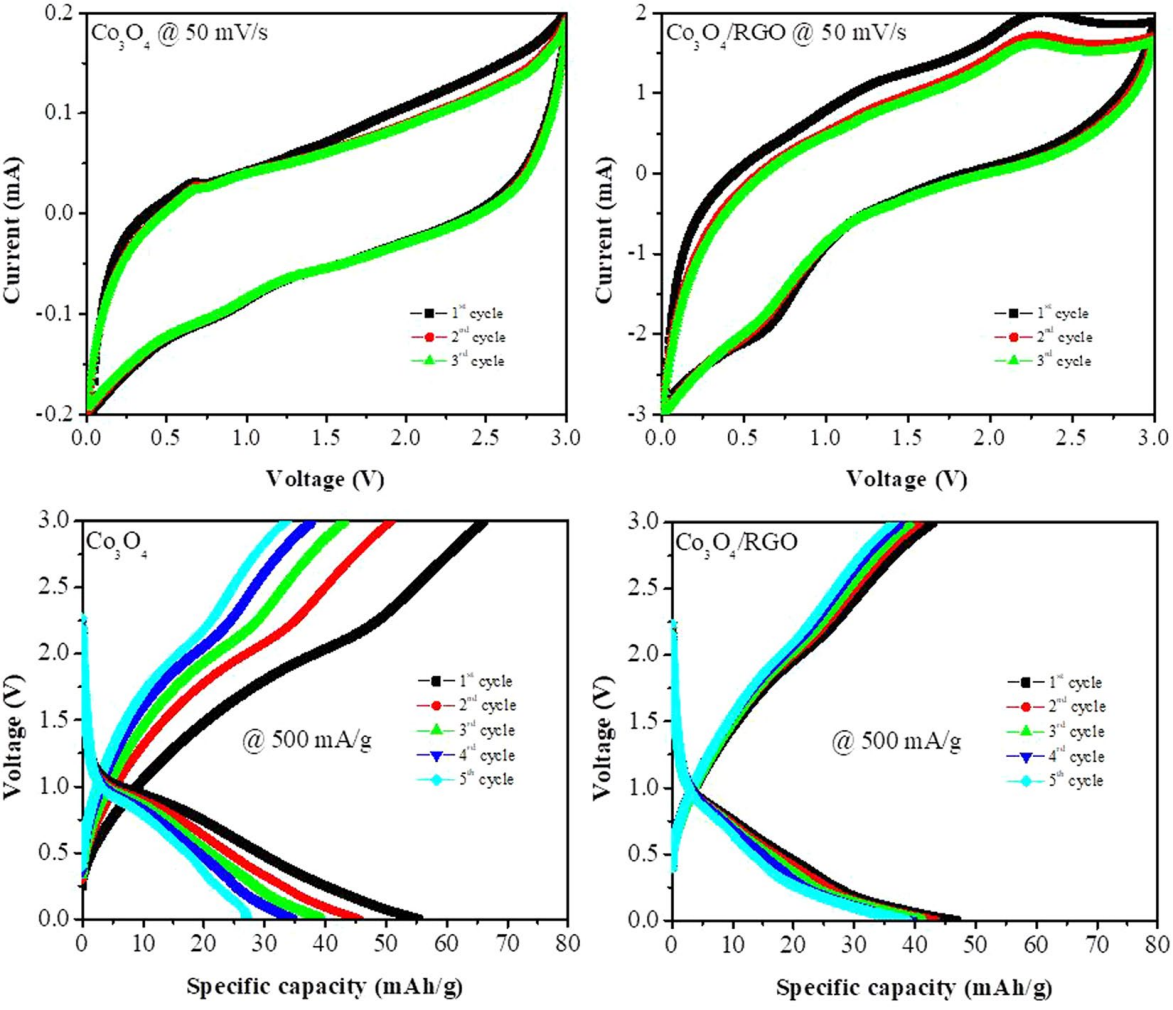
Cyclic voltammetric (CV) and galvanostatic charge-discharge curves of Co_3_O_4_ and Co_3_O_4_/RGO nanocomposites.

To study the electrochemical behavior of the Co_3_O_4_ nanoparticles and Co_3_O_4_/RGO nanocomposites, CV was first performed in a range of 0 and 3.0 V at a scan rate of 50 mV/s for 3 cycles, as shown in Fig. 3. A cathodic or reduction peak appeared at approximately 0.87 V for the Co_3_O_4_ nanoparticles and at 0.67 V for the Co_3_O_4_/RGO nanocomposites in the first scan. These peaks resulted from the reduction of Co_3_O_4_ to Co metal, the formation of clusters between Co and Li_2_O, the insertion of lithium into RGO in the case of the Co_3_O_4_/RGO nanocomposites and the formation of a solid electrolyte interphase (SEI) layer on the active material^9^. In the anodic or oxidation scan, two peaks at 1.26 V and 2.28 V for the Co_3_O_4_/RGO nanocomposites were observed after the first cycle; these peaks are due to the de-insertion of lithium ions from RGO and the reversible oxidation of Co metal to Co_3_O_4_^29,30^, respectively. However, a weak or almost nonexistent oxidation peak was observed for Co_3_O_4_, which could be due to the high scan rate. No significant drop in the peak intensity in subsequent cycles was observed for either the Co_3_O_4_ nanoparticles or Co_3_O_4_/RGO nanocomposites, suggesting the good reversibility of lithium storage and a high stability^11^. The electrochemical conversion reaction of Co_3_O_4_-based anodes can be described as^10^:

$${{\rm{Co}}}_{{\rm{3}}}{{\rm{O}}}_{{\rm{4}}}+8\,{\rm{Li}}\leftrightharpoons 4\,{{\rm{Li}}}_{2}{\rm{O}}+3{\rm{Co}}$$

Also, the Co_3_O_4_/RGO nanocomposite displayed a higher current than the Co_3_O_4_ nanoparticles, which was due to the incorporation of conductive RGO sheets.

Typical charge/discharge cycling of the prepared Co_3_O_4_ nanoparticles and Co_3_O_4_/RGO nanocomposites was performed at a current density of 500 mA/g for five cycles, as shown in Fig. 3. The initial charge/discharge capacities were approximately 66/55.5 mAh/g and 47/42.7 mAh/g for the Co_3_O_4_ nanoparticles and Co_3_O_4_/RGO nanocomposites, respectively, at the same current density. The Co_3_O_4_/RGO nanocomposites exhibited stable cycling performance with 85% capacity retention after the 5^th^ cycle and charge/discharge capacities of 39/36.5 mAh/g, while the Co_3_O_4_ nanoparticles showed a dramatic capacity loss with only 48% capacity retention after 5 cycles, evidencing the positive effect of the RGO sheets that led to an enhanced electrochemical response.

**Figure 4:**
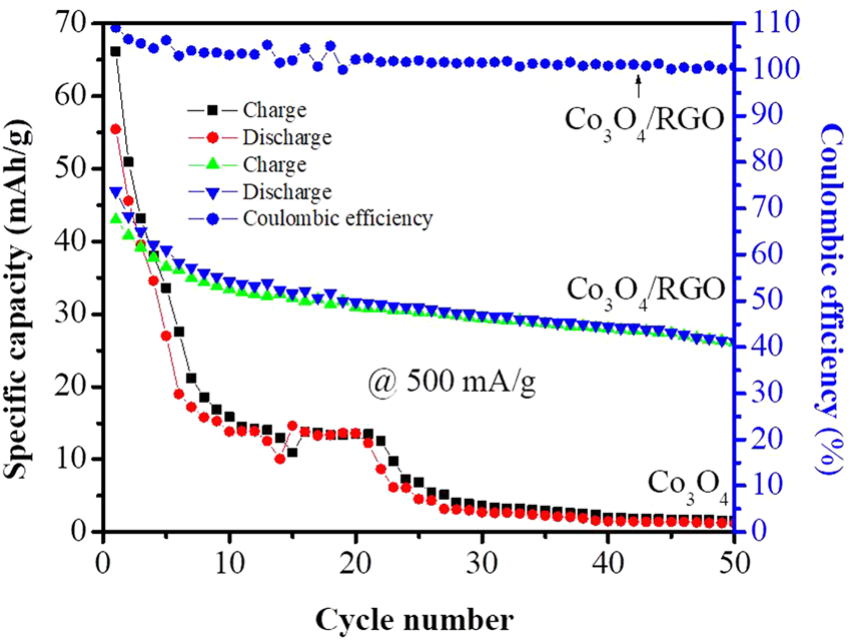
Cycling performance of Co_3_O_4_ nanoparticles and Co_3_O_4_/RGO nanocomposites at 500 mA/g for 50 cycles.

**Figure 5:**
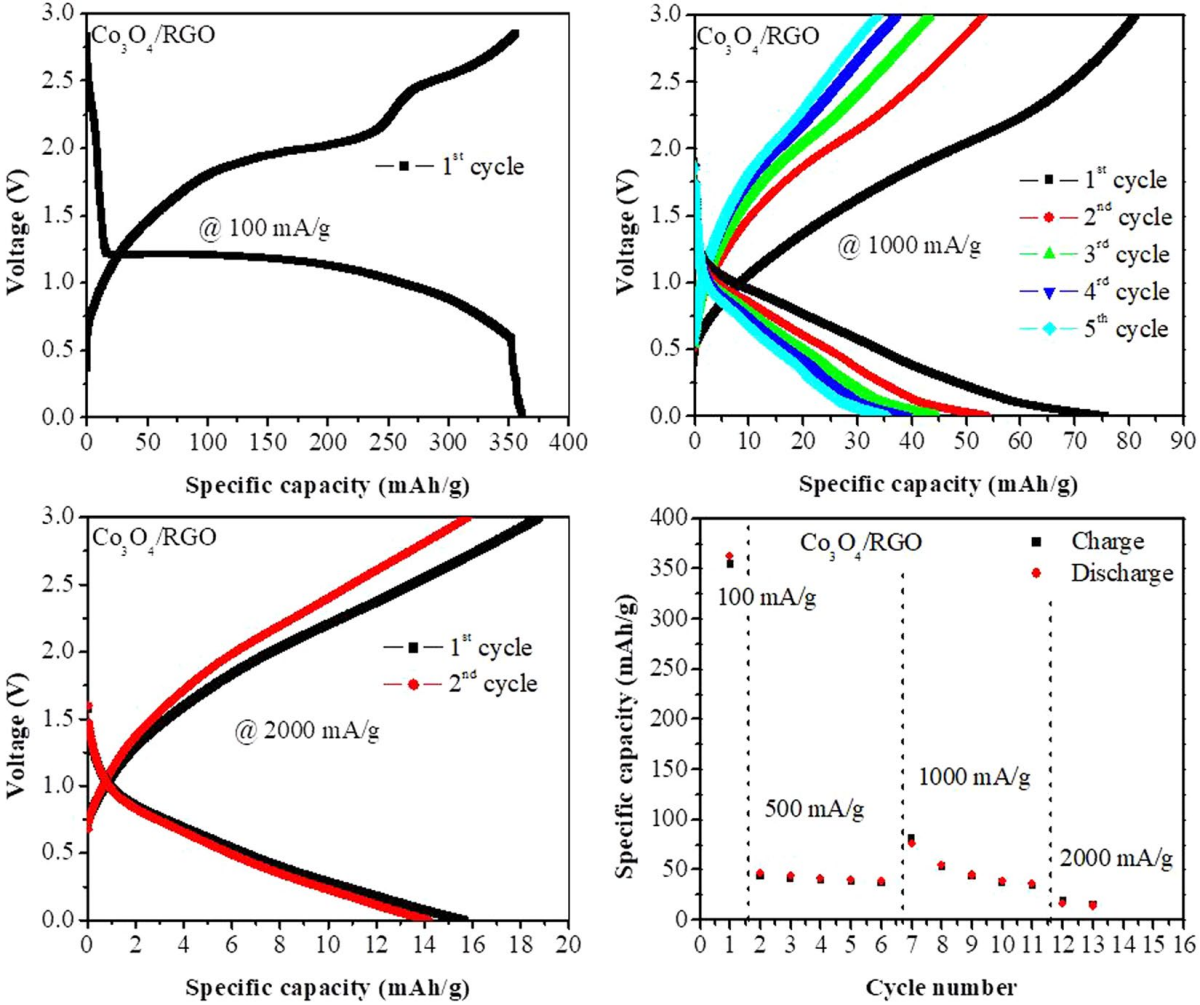
Rate capabilities of Co_3_O_4_ nanoparticles and Co_3_O_4_/RGO nanocomposites.

To further investigate the stability of the Co_3_O_4_ nanoparticles and Co_3_O_4_/RGO nanocomposites, the cyclic performance and rate capability were evaluated, as shown in Figs 4 and 5. The Co_3_O_4_/RGO nanocomposites showed a more stable cyclic perfomance than Co_3_O_4_ with a reversible charge/discharge capacity of 26 mAh/g after the 50^th^ cycle, while the discharge capacity of Co_3_O_4_ dropped continuously with each cycle, giving a reversible capacity of only 1.6/1.2 mAh/g after the 50^th^ cycle. Fig. 4 also shows the columbic efficiency of the Co_3_O_4_/RGO nanocomposites. During the first cycle, the Co_3_O_4_/RGO nanocomposites exhibited a coulombic efficiency above 100% due to the occurrence of a reverse reaction that involved the embedding of Li_2_O in the metal particles, which enhanced the electrochemical activity as a result of Li_2_O decomposition and metal–oxygen bond formation^31,32^. Thus, this metal could be oxidized to higher valence states, causing the delithiation capacity to be higher than the lithiation capacity, which resulted in a coulombic efficiency above 100%. The coulombic efficiency of the Co_3_O_4_/RGO nanocomposites was 100% after 50 cycles (Fig. 4), while the Co_3_O_4_ nanoparticles exhibited a coulombic efficiency of 75% after 50 cycles (Fig. S1).

The rate capability of the Co_3_O_4_/RGO nanocomposites is shown in Fig. 5. At a low current density (100 mA/g), the Co_3_O_4_/RGO nanocomposites displayed initial charge/discharge capacities of 358/363 mAh/g, and when a higher current density was used (1000 mA/g), the charge/discharge capacities were 82.5/77.7 mAh/g.

**Figure 6:**
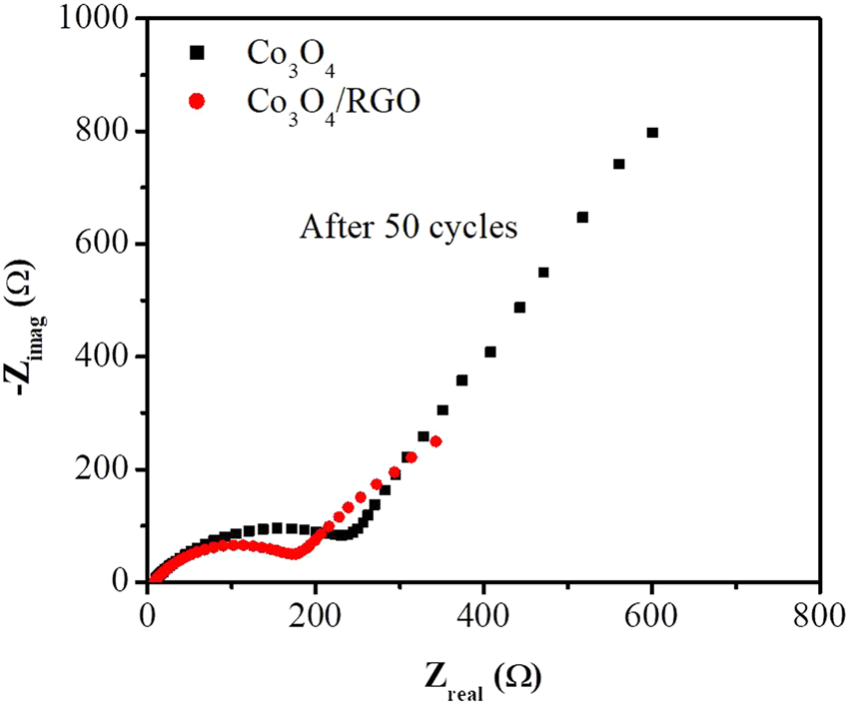
Nyquist plots of Co_3_O_4_ nanoparticles and Co_3_O_4_/RGO nanocomposites.

EIS studies were performed for both the Co_3_O_4_ nanoparticles and Co_3_O_4_/RGO nanocomposites, as shown in the Nyquist plots in Fig. 6. The semicircle in the high-medium frequency region is due to the charge-transfer resistance. The diameter of the semicircle for Co_3_O_4_/RGO is smaller than that for Co_3_O_4_, which indicates that Co_3_O_4_/RGO is capable of faster charge transfer and that it exhibits less internal electrochemical resistance than Co_3_O_4_. The sloped lines in the low-frequency region can be attributed to the mass transfer process or the Warburg resistance. The steeper tail for the Co_3_O_4_/RGO nanocomposites indicates a lower ion diffusion resistance and enhanced mass transport compared to Co_3_O_4_. Thus, the Co_3_O_4_/RGO nanocomposites showed high electrical conductivity and rapid charge and mass transfer, which play critical roles in the overall battery performance^33,34^.

**Figure 7:**
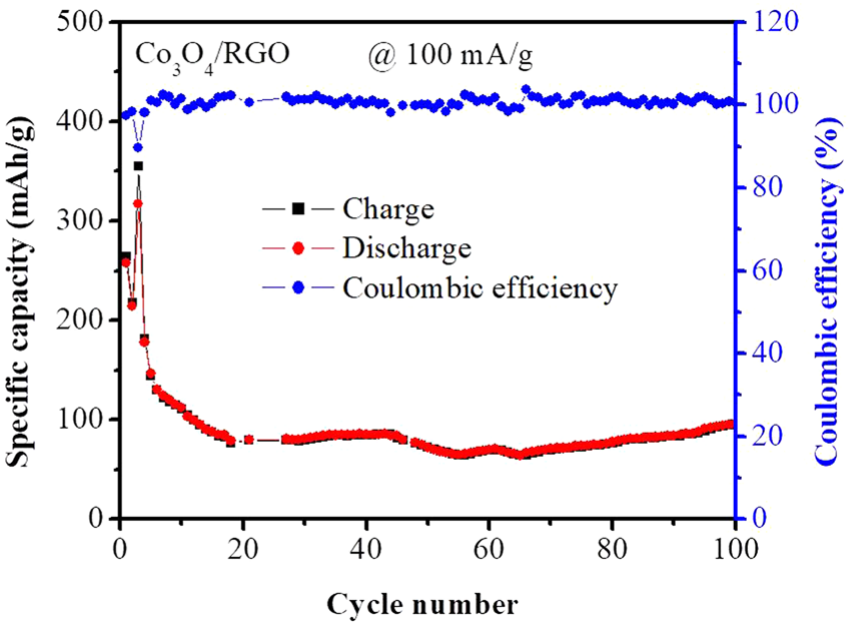
Cycling performance of Co_3_O_4_/RGO nanocomposites at 100 mA/g for 100 cycles.

Microwave-assisted techniques has been widely used for the synthesis of graphene based metal oxides as electrodes in lithium-ion batteries which includes Co_3_O_4_-graphene^35–37^, CuO-graphene^38^, Fe_x_O_y_-graphene^39–41^, Mn_3_O_4_-graphene^42,43^, SnO_2_-graphene^44–48^ and ZnO-graphene^49^ nanocomposites. As compared with the results in literature^35–49^ shown in Table 1, the Co_3_O_4_/RGO nanocomposites displayed a good reversible capacity of 96.36 mAh/g at 100 mA/g after 100 cycles with the addition of only 1% graphene content as shown in Fig. 7 which was conducted for a new coin cell with Co_3_O_4_/RGO nanocomposites as electrodes. Also, CV at low current and EIS after 100 cycles were also performed as shown in Figs S2 and S3.

**Table 1:** Comparison of graphene content and electrochemical performance of Co_3_O_4_/RGO nanocomposites with Co_3_O_4_/graphene and other metal oxides/graphene reported.

Nanocomposites	Synthesis method	Graphene content (%)	Reversible capacity (mAh/g)	Current density (mA/g)	Cycle number (nth)	References
Co_3_O_4_/RGO	Microwave irradiation (microwave oven)	1	96.36	100	100	This work
Co_3_O_4_-graphene sheet-on-sheet nanocomposite	Microwave-assisted	46.2	1065	89	30	^35^
Co_3_O_4_ quantum dots/graphene	Microwave-assisted (microwave oven)	40	1785	89	90	^36^
Co_3_O_4_-graphene sheet-on-sheet nanocomposites	Microwave-assisted (microwave oven)	18.4	1036.9	100	50	^37^
Graphene-wrapped CuO	Microwave-assisted hydrothermal	16.9	349	1000	60	^38^
RG-O/Fe_2_O_3_ composite	Microwave irradiation (microwave oven)	20	1027	100	50	^39^
α-Fe_2_O_3_/RGO nanocomposites	Microwave-assisted	21.3	650	1000	50	^40^
Fe_3_O_4_-ONCs@rGO	Microwave assisted (microwave oven)	43	540	100	120	^41^
Mn_3_O_4_–graphene	Microwave hydrothermal	26.72	900	45	50	^42^
Mn_3_O_4_/graphene	Microwave hydrothermal	13.47	500	60	100	^43^
SnO_2_-Graphene	Microwave autoclave	33.3	590	100	200	^44^
Graphene-SnO_2_	Microwave-assisted (microwave oven)	30	890	500	80	^45^
Graphene-SnO_2_	Microwave irradiation (microwave oven)	35.6	1000	50	100	^46^
SnO_2_/graphene	Microwave hydrothermal	10.03	978.6	200	100	^47^
2D SnO_2_/graphene	Microwave-assisted	10	688.5	200	500	^48^
ZnO@Graphene	Microwave-assisted (microwave oven)	50	460	700	50	^49^

**Figure 8:**
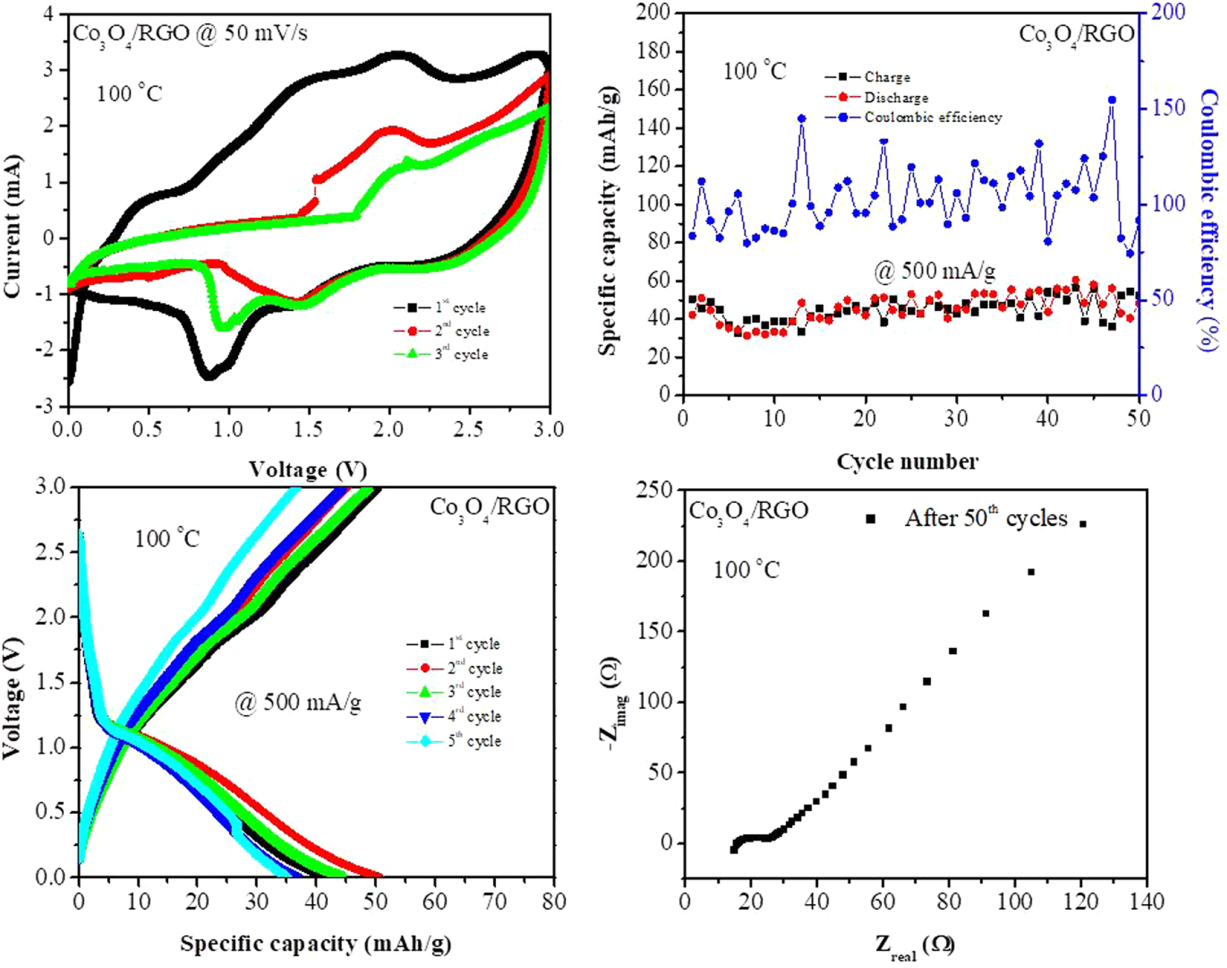
Cyclic voltammetric (CV), galvanostatic charge/discharge curves, cycling performance and EIS spectra of Co_3_O_4_ nanoparticles and Co_3_O_4_/RGO nanocomposites performed at 100 °C.

Further electrochemical investigations were performed to test the electrochemical performance of the Co_3_O_4_/RGO nanocomposites at a higher operating temperature of 100 °C. Figure 8 shows the CV curve of the Co_3_O_4_/RGO nanocomposites performed at 100 °C in the range between 0 and 3.0 V at a scan rate of 50 mV/s for 3 cycles. The Co_3_O_4_/RGO nanocomposites exhibited an ideal CV curve of a Co_3_O_4_-based anode at 100 °C, with two reduction peaks at 0.86 V and 1.45 V in the first scan. These peaks resulted from the reduction of Co_3_O_4_ to Co metal, the formation of clusters between Co and Li_2_O, the insertion of lithium into RGO and the formation of an SEI layer on the active material^9^. Two peaks were observed at 1.45 V and 1.96 V in the anodic scan after the first scan, and these peaks are due to the de-insertion of lithium ions from RGO and the reversible oxidation of Co metal to Co_3_O_4_^29,30^, respectively. The overlap between the second and the third cycles indicates the enhanced cycling stability of the Co_3_O_4_/RGO nanocomposites^50^. Furthermore, the current response of Co_3_O_4_/RGO increased with stronger and sharper peaks when the operating temperature was 100 °C, which indicates the role of the high temperature in enhancing the CV performance of the Co_3_O_4_/RGO nanocomposites.

The galvanostatic charge/discharge capacities of the Co_3_O_4_/RGO nanocomposites measured at an operating temperature of 100 °C with a current density of 500 mA/g for 5 and 50 cycles are shown in Fig. 8. No decrease of charge/discharge capacity was observed when the operating temperature was increased from ambient (Fig. 4) to 100 °C (Fig. 8) at a constant current density, with 100% capacity retention for 50 cycles and a coulombic efficiency of 100%. Note that a coulombic efficiency above 100% was observed, which was due to the reversible insertion of Li_2_O into the metal particles.

To further investigate the electrochemical behavior of the Co_3_O_4_/RGO nanocomposites at high operating temperature, EIS tests were conducted, as shown in Fig. 8. Comparing the Nyquist plots of Co_3_O_4_/RGO (Fig. 6) at room temperature and 100 °C (Fig. 8), the semicircle decreased in the high-mid frequency region following the increase of temperature to 100 °C, exhibiting a reduction in the electrochemical resistance, which was due to the larger amount of charges that could overcome the activation energy, thereby reducing the internal electrochemical resistance. In addition, the increase in the slope of the straight line of Co_3_O_4_/RGO in the low frequency region of the spectrum measured at 100 °C (Fig. 8) relative to that measured at room temperature (Fig. 6) is an indication of faster mass transport. This suggests the occurrence of excellent electrochemical behavior at high temperatures, which matches with the above CV and charge/discharge tests performed at a high temperature of 100 °C^51^.

## Conclusions

We successfully prepared Co_3_O_4_ nanoparticles and Co_3_O_4_/RGO nanocomposites via a microwave-assisted route. FTIR spectroscopy confirmed the formation of graphene-based Co_3_O_4_ nanoparticles. Co_3_O_4_/RGO nanocomposites’ thermal stability was determined using TGA. Morphological studies using TEM further confirmed the formation of Co_3_O_4_ nanoparticles in addition to Co_3_O_4_ nanoparticles supported on RGO. The prepared Co_3_O_4_/RGO nanocomposites showed excellent electrochemical behavior as anodes in li-ion batteries. A superior electrochemical response which includes enhanced charge/discharge capacity and cycling stability was observed. The enhanced electrochemical performance relative to that of the pure Co_3_O_4_ nanoparticles, even when high current densities are applied, is attributed to the incorporation of 2D graphene, which resulted in a surface area almost four times larger than that of pure Co_3_O_4_, and to the exfoliation and good integrity of the RGO sheets in the Co_3_O_4_/RGO nanocomposites, as determined from chemical and thermal studies. Furthermore, increasing the operating temperature from ambient to 100 °C further enhanced the electrochemical performance, making the prepared nanocomposites potential for high temperature li-ion batteries.

## Supplementary information

Enhanced Electrochemical performance at high temperature of Cobalt Oxide/Reduced Graphene Oxide Nanocomposites and its application in lithium-ion batteries
