# Supplementary material for: Enhanced Electrochemical performance at high temperature of Cobalt Oxide/Reduced Graphene Oxide Nanocomposites and its application in lithium-ion batteries
Source: Sci Rep. 2019 Jan 10;9:44. doi: 10.1038/s41598-018-37032-5 (PMC6328569; doi:10.1038/s41598-018-37032-5)
Supplement: Supplementary file 1 — Enhanced Electrochemical performance at high temperature of Cobalt Oxide/Reduced Graphene Oxide Nanocomposites and its application in lithium-ion batteries [file 41598_2018_37032_MOESM1_ESM.docx]

**Enhanced Electrochemical performance at high temperature of Cobalt Oxide/Reduced Graphene Oxide Nanocomposites and its application in lithium-ion batteries**

Yasmin Mussa^1^, Faheem Ahmed^1^, Hatem Abuhimd^2^ Muhammad Arsalan^3^, Edreese Alsharaeh*^1^

^1^College of Science and General Studies, Alfaisal University, P.O. Box 50927, Riyadh, 11533, Saudi Arabia

^2^National Nanotechnology Center, King Abdulaziz City for Science and Technology, P.O. Box 6086, Riyadh 11442, Saudi Arabia

^3^EXPEC Advanced Research Center, Saudi Aramco, P.O. Box 5000, Dhahran, 31311, Saudi Arabia

*[ealsharaeh@alfaisal.edu](mailto:ealsharaeh@alfaisal.edu)

Figure S1: Coulombic efficiency of the Co_3_O_4_ nanoparticles over 50 cycles.

Figure S2: CV of the Co_3_O_4_/RGO nanocomposites at 0.1 mV/s.

Figure S3: Nyquist plots of the Co_3_O_4_/RGO nanocomposites after 100 charge/discharge cycles performed at 100 mA/g.
